# Neuroprotective role of morin hydrate on 3-nitropropionic acid-elicited huntington's disease: in vivo investigation of RIPK1/RIPK3/MLKL necroptosis signaling pathway

**DOI:** 10.1186/s10020-025-01172-y

**Published:** 2025-04-11

**Authors:** Eman M. Elbaz, Rabab H. Sayed, Amany A. Abdelkader, Atef Tadros Fahim

**Affiliations:** 1 Department of Biochemistry, Faculty of Pharmacy, Cairo University, Kasr El Aini St, Cairo, 11562 Egypt; 2 Department of Pharmacology and Toxicology, Faculty of Pharmacy, Cairo University, Cairo, 11562 Egypt; 3 School of Pharmacy, Newgiza University, Giza, Egypt

**Keywords:** Huntington’s disease, Morin hydrate, Necroptosis, Necrosulfonamide, 3-nitropropionic acid

## Abstract

**Background:**

Huntington’s disease (HD) is a rare dominantly inheritable autosomal neurodegenerative disease with unclear pathophysiological pathways. In neurodegenerative disorders, including HD, necroptosis plays a significant role in neuronal death. Morin hydrate (MH), a natural bioactive flavonoid, has various pharmacological properties via orchestrating neuroinflammation, apoptosis, and necroptosis. Up to now, there is no extant data on the impact of MH on the necroptotic pathway in HD.

**Aim:**

This research aimed to scrutinize the effect of MH on neurodegeneration initiated by 3-nitropropionic acid (3-NP) administration in rats via modulating necroptosis and apoptosis signaling pathways and compare it with necrosulfonamide (NSA) as a necroptosis inhibitor.

**Methods:**

HD was triggered in male wistar rats by intraperitoneal injection of 3-NP (10 mg/kg/day) for 14 days. Intraperitoneal injection of MH (20 mg/kg/day, i.p.) or NSA (1.65 mg/kg/day, i.p.) an hour prior to 3-NP administration for 14 days. At the end of study, rats were weighed, and their locomotor activity was assessed via grip strength and open field tests. Striata of rats were investigated histologically and immunohistochemically by evaluation the expression levels of glial fibrillary acidic protein (GFAP). Striatal tumor necrosis factor-alpha (TNF-α), caspase 3, and 8 levels were quantified through the ELISA technique, while striatal expression of necroptosis-associated proteins; phosphorylated form of receptor interacting protein kinase 1/3(p-RIPK1, p-RIPK3) and phosphorylated form of mixed lineage kinase domain-like protein (p-MLKL) were assessed by the Western blot technique. Striatal succinate dehydrogenase (SDH) activity was assayed colorimetrically. Finally, gene enrichment analysis using ShinyGO was employed.

**Results:**

MH and NSA significantly mitigated body weight loss and ameliorated locomotor deterioration, besides reversing histological abnormalities in the striatum of rats. Intriguingly, MH exerted similar effects on specific biomarkers and molecular signals as NSA. MH and NSA inhibited neuroinflammation, apoptosis, and necroptosis by significantly decreasing the striatal (TNF-α), caspase 3, and necroptosis-associated proteins (P-RIPK1, P-RIPK3, and P-MLKL) levels. Besides, MH and NSA also decreased striatal GFAP and increased SDH activity. Gene enrichment analysis revealed a significant interaction between genes. Together, MH exerts a neuroprotective action on 3-NP-elicited HD rats via reducing neuroinflammation, apoptosis, and necroptosis. This study highlights MH as a potential protection against HD, calling for further research to confirm its neuroprotective effects.

## Introduction

Huntington’s disease (HD) is a dominant autosomal disease distinguished by neurodegeneration of the central nervous system (Novak and Tabrizi 2010). HD originates from unstable expansion of CAG trinucleotide repetitions (> 35) in the huntingtin (HTT) gene on chromosome 4 (Bano et al. 2011). The most common clinical manifestations of HD are uncontrolled excessive motor movements called chorea, psychiatric symptoms, and cognitive decline (Finkbeiner 2011). HD’s exact pathophysiological pathways are not well known; nevertheless, studies using transgenic animal models of the disorder are shedding light on the disorder’s contributing components and possible therapies (Walker 2007).

3-Nitropropionic acid (3-NP) is a natural toxin which can cross the blood-brain barrier (BBB) (Túnez et al. 2010). The neurotoxicity of 3-NP is mainly due to the mitochondrial disruption evoked by the toxin and the associated generation of reactive oxygen species, which trigger cell death pathways (Nasr et al. 2003). 3-NP specifically induces striatal degeneration (Borlongan et al. 1997). Animals exposed to 3-NP have been shown to exhibit morphological, behavioral, and biochemical alterations like those seen in HD (Túnez et al. 2010). These changes may be linked to three factors implicated in the pathogenesis of HD: (i) impaired energy metabolism, defined by mitochondrial dysfunction; (ii) excitotoxicity, in which striatal neurons exhibit higher sensitivity to glutamatergic activation by N-methyl-D-aspartate receptors, which results in excitotoxic neurodegeneration; and (iii) oxidative stress (Sorolla et al. 2012).

Apoptosis and necrosis are the two main forms of cell death that are essential in the emergence of many diseases (Healy et al. 1998). Historically, apoptosis is known as programmed cell death as it could be controlled, while necrosis is known as accidental cell death as it could not be regulated (Berghe et al. 2014). In another study, apoptosis is called caspase-dependent cell death, as caspase activation is essential in the execution of apoptosis, while necrosis is known as caspase-independent cell death (Holler et al. 2000). According to previous research, necrosis has a regulated caspase-independent form called necroptosis (Zhang et al. 2017). Unlike apoptosis, more recently identified forms of regulated cell death, such as necroptosis, ferroptosis (iron-dependent regulated form of cell death)(Hirschhorn and Stockwell 2019), and pyroptosis (caspase 1-dependent programmed cell death) (Bergsbaken et al. 2009), have gained significant attention. We focused on necroptosis, as several investigations have uncovered the relationship between the necroptosis pathway and neurodegenerative diseases such as ischemic stroke (Zhang et al. 2019), Alzheimer’s disease (AD) (Motawi et al. 2020; Abdelhady et al. 2023) and Parkinson’s disease (PD) (Oñate et al. 2020). Zhu et al. investigated the role of necroptotic signaling in HD pathophysiology, which was confirmed by the therapeutic benefit of the necroptosis inhibitor, necrostatin-1, against HD-associated neuropathologies (Zhu et al. 2011).

Necroptosis is a novel type of programmed cell death which shows the morphology of both apoptosis and necrosis and is frequently induced where the apoptotic pathway is blocked (Dhuriya and Sharma 2018). In addition, it is easily distinguished from apoptosis as it is not accompanied by important apoptosis regulators like caspases, B-cell lymphoma 2 protein (Bcl-2), or the release of cytochrome c from mitochondria (Degterev et al. 2008). Moreover, necroptosis can act as a backup mechanism for apoptosis-resistant conditions, which can overcome the common but complex problem in clinical cancer therapy (Wu et al. 2020). Receptor-interacting protein kinases 1 and 3 (RIPK1 and RIPK3) induce the development of a cytosolic signaling complex termed the necrosome, which promotes necroptosis by serving as signaling mediators to activate the mixed lineage kinase domain-like protein (MLKL) (Linkermann and Green 2014). MLKL was crucial in necroptosis, which led to leakage in the cell membrane and cytokine release (Wu et al. 2020). Several factors can induce necroptotic cell death, including the binding of tumor necrosis factor (TNF), cytokine and membrane receptors, interferons (IFNs) stimulation, or viral infection (Wu et al. 2020).

TNF binding activates TNF receptor-1 (TNFR-1), triggering a sequence of actions that control apoptosis and necroptosis. Procaspase-8 can also be detected in necrosomes and have a crucial role in the regulation of necroptosis and apoptosis. When procaspase-8 binds with cellular FLICE (FADD-like IL-1β-converting enzyme)-inhibitory protein (c-FLIP) long (c-FLIPL), it is activated and exerts adequate proteolytic activity to dissociate the RIP1-RIP3 complex, hence inducing apoptosis and suppressing necroptosis. But when procaspase-8 binds with c-FLIP short (c-FLIPS), it is not activated and permits the formation of RIP1-RIP3 complex, which promotes necroptosis (Zhang et al. 2017; Park et al. 2021).

Necrosulfonamide (NSA), a necroptotic blocker, inhibits the phosphorylation of MLKL, which is an essential necroptotic executioner (Linkermann and Green 2014). Several studies have linked necroptosis to the pathogenesis of various neurodegenerative disorders, including multiple sclerosis (Ofengeim et al. 2015), AD (Motawi et al. 2020) and PD (Wu et al. 2015). From this point of view, using small-molecule modulators to inhibit the necroptosis pathway is emerging as an efficient approach in HD therapy.

Morin hydrate (MH) (3,5,7,2’,4’-pentahydroxyflavone) is a natural polyphenol bioactive compound that was originally isolated from *Moraceae* family such as onion, seaweeds, Indian guava (Psidium guajava), figs (Chlorophora tinctoria), almond (Prunus dulcis) and Osage orange (Rajput et al. 2021). It demonstrates a broad spectrum of pharmacological and biological features, including anticancer, anti-inflammatory, chemoprotective, and antioxidant activities (Bachewal et al. 2018). More importantly, morin has low cytotoxicity, and its safety has been well-established by a previous study (Rajput et al. 2021). In addition, it can cross the BBB (Youdim et al. 2004). Morin suppresses neuroinflammation, neuronal apoptosis, and oxidative stress, demonstrating a typical neuroprotective activity against neurotoxicity (Zhang et al. 2010). It was previously demonstrated that morin may reduce neuronal damage in AD (Mohammadi et al. 2021) and HD(Mohamed et al. 2023; El-Emam et al. 2024). Furthermore, it was reported that morin exerted a neuroprotective effect in vitro and in vivo PD models (Zhang et al. 2010).

In our research, we aimed to scrutinize the possible neuroprotective actions of MH against the 3-NP-elicited HD rat model by modulating the necroptosis and apoptosis pathways and to compare MH with NSA as a necroptosis inhibitor.

### Materials and methods

### Chemicals and drugs

Both 3-NP (Cat. No. 504-88-1 ) and MH ( Cat. No. 654055-01-3) were obtained from Sigma-Aldrich Co. (St Louis, MO, USA). 3-NP was dissolved in 0.9% saline in a volume of 0.2 mL 200 g-1 animal body weight(Elbaz et al. 2019; Mohamed et al. 2023) while MH dissolved in < 0.1% dimethyl sulfoxide (DMSO)(Zhang et al. 2010; Al Numair et al. 2012). NSA (Cat. No. 480073) was acquired from Merck Calbiochem (Darmstadt, Germany), dissolved < 0.1% DMSO, and diluted in saline(Jiao et al. 2019; Motawi et al. 2020). All chemicals in this study were of high analytical quality.

### Animals

We procured forty male adult Wistar albino rats, weighing between 180 and 200 g, from the National Research Center located in Cairo, Egypt, and housed them (4/cage) in the animal house facility of the Faculty of Pharmacy, Cairo University, Cairo, Egypt under the appropriate experimental conditions (temperature: 25 ± 2ºC, humidity range: 60–70% and 12 h/12 h light/dark cycle). All through the experiment, rats were given unrestricted access to food and water. One week prior to the study, rats were acclimated to the laboratory conditions. All rats were weighed in grams in the beginning of the study. All procedures followed the “Research Ethical Committee” guidelines (permit number: 3059) of the Faculty of Pharmacy, Cairo University (Cairo, Egypt). All possible attempts were made to lessen the animals’ pain or discomfort.

### Experimental design

**Fig. 1:**
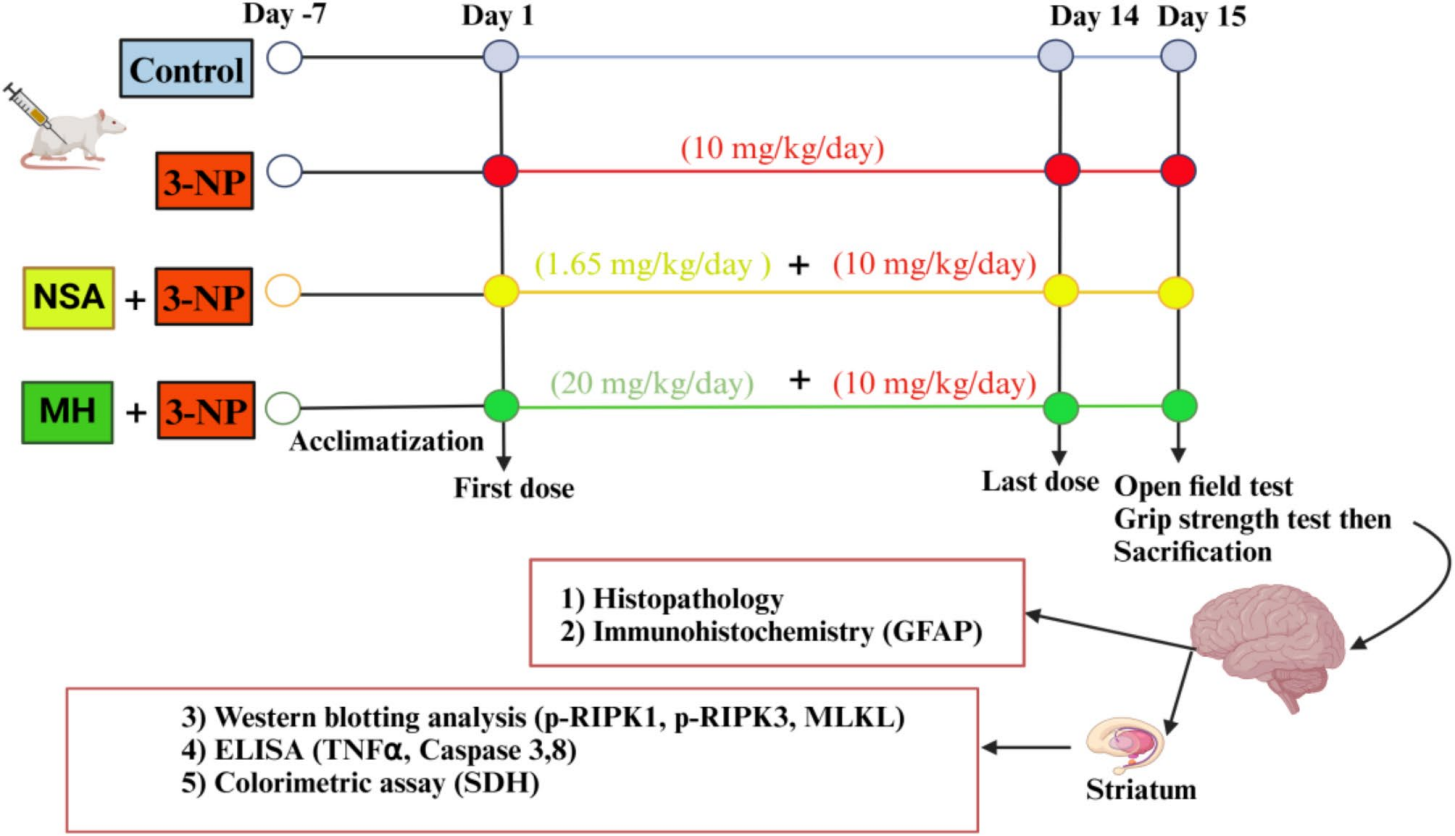
Schematic diagram of the study design. ELISA: Enzyme-linked immunosorbent assay, GFAP: Glial fibrillary acidic protein, MH: morin hydrate, MLKL: mixed lineage kinase domain-like protein, 3-NP: 3-nitropropionic acid, NSA: necrosulfonamide, RIPK1/3: receptor interacting protein kinase 1/3, SDH: succinate dehydrogenase, TNF-α: tumor necrosis factor-alpha. Created with https://www.BioRender.com

In this study, forty rats were randomly distributed to each of four groups (*n* = 10 per group), where the first group represented the control in which rats injected with < 0.1% DMSO (1 ml/kg/day) diluted in 0.9% normal saline intraperitoneally (i.p.) for fourteen days. Rats in the second group (3-NP) were induced for HD by i.p. injection of freshly prepared 3-NP in 0.9% saline (10 mg/kg/day) (Elbaz et al. 2019) for fourteen days. In the third (NSA + 3-NP) and fourth (MH + 3-NP) groups, the rats were pretreated with NSA that was dissolved in DMSO and diluted with normal saline (1.65 mg/kg/day, i.p.) (Motawi et al. 2020), and MH dissolved in < 0.1% DMSO (20 mg/kg/day, i.p.)(Mohammadi et al. 2021), respectively, 1 h before 3-NP (10 mg/kg/day, i.p.) administration for 14 days (Fig. 1).

### Behavioral evaluation

Animals underwent behavioral tests 24 h after the last 3-NP injection with at least thirty min between each test. All tests were performed throughout the animal’s light cycle.

#### Open field test

An open-field test was applied to evaluate the spontaneous locomotor activity. The apparatus consisted of a squared wooden arena with a red-painted wall measuring 80 cm in width, 80 cm in length, and 40 cm in height. White lines were used to divide the floor into 16 squares of equal size. The apparatus was placed in a soundless room and under white light. Animals were individually positioned in the middle of the arena, and an overhead video tracking system was used to record the locomotor behavior for 3 min. Any feces or urine was removed after each rat was tested, and the arena was wiped with 20% ethyl alcohol. Total distance travelled (the total distance that the animal travelled during the test), mean speed (the average speed of the animal during a test), time immobile (the amount of time the animal was immobile during the test), immobile episodes (the number of times the animal was immobile during the test), and immobile latency (the latency to the start of the first moment in the test when the animal is considered to be immobile) were recorded for each rat using ANY-maze software (Flaisher-Grinberg and Einat 2009).

#### Grip strength test

Grip strength meter (Model 47200, Ugo Basile, Comerio, Italy) was employed for evaluation of the upper limb impairment. One rat at a time was placed near the T-bar, and the grip strength meter was set to start recording. Once the rat positioned both of its forepaws on the T-bar, it was slowly pulled away from the T-bar by their tail in a parallel direction to the work surface. The maximum force exerted on the T-bar was recorded in grams when the rat released its grip. Three tests were performed for each rat, and the mean was calculated (Innes 1999).

### Brain processing

At the end of the study period (14th day), and after completing all behavioral assessments, all rats were weighed in grams to calculate % change in body weight, then slaughtered via cervical dislocation under light anesthesia utilizing thiopental (5 mg/kg, i.p) (Onk et al. 2016). Brains were rapidly removed, followed by rinsing with ice-cold saline. The brains of the aforementioned groups were separated into two groups. One group (*n* = 3) was immersed in 10% (v/v) formalin for 24 h to apply hematoxylin and eosin (H&E) staining, along with immunostaining, to investigate histopathological changes and perform immunohistochemical analysis. In the other group (*n* = 7), the striatum was removed from all brains and placed on an ice-cold eppendorf tubes to perform the biochemical analysis.

### Histopathological examination

#### Hematoxylin and Eosin staining

H&E staining was conducted to estimate the neuronal damage in striatal tissues. After removing brain tissues from rats, brains were washed with ice-cold saline and preserved with neutral buffered 10% formalin for 24 h. Samples were desiccated by incubating in sequential dilutions of ethanol, cleared in xylene, and immersed in paraffin to demonstrate striatal regions. The embedded brain tissues were serially coronal sectioned (4 μm thickness) by rotatory microtome and fixed on glass slides. After that, the brain slices were inspected using the light microscope after being dyed with H&E. Morphological changes, including congested blood vessels, perivascular lymphocytic cuffing, gliosis, and degenerated neurons, were defined in each section. Histological lesions were graded on a scale from 0 to 4 as follows: (0) normal histology with no alterations, (1) mild changes affecting less than 25%, (2) moderate changes involving 25–50%, (3) severe changes covering 50–75%, and (4) extensive tissue damage exceeding 75% (Avallone et al. 2021).

#### Glial fibrillary acidic protein (GFAP) immunostaining

A rat monoclonal antibody (Thermo Fisher Scientific Inc., Rockford, IL, USA) was used for immunohistochemical staining of GFAP. Every step was carried out as directed by the manufacturer’s guidelines. The GFAP immunoreactivity proportion was determined in 5 randomly selected distinct areas per tissue slice under inspection of a Full HD microscopic camera controlled by the Leica application module for tissue section analysis (Leica Microsystems GmbH, Wetzlar, Germany)(Ezzat et al. 2022).

### Biochemical parameters

The right-side striatum was homogenized in ice-cold saline to form 10% homogenates, which were divided into several aliquots for the Enzyme-linked immunosorbent assay (ELISA), which was used to assess TNF-α, caspase 3/8 and for colorimetric assay of succinate dehydrogenase (SDH). The left-side striatum was kept at -80 ºC to be used for Western blot analysis.

**2.7.1. Enzyme-linked immunosorbent assay** (**ELISA)**: Striatal TNF-α, caspase 3, and 8 levels were quantified through the ELISA technique. Rat-specific commercial ELISA kits were employed as directed by the manufacturer’s guideline to determine the striatal levels of the following biomarkers: TNF-α (Cat. No. SEA133Ra, Cloud-Clone Corp, Huston, TX, USA), caspase 3 (Cat. No. SEA626Ra, Cloud-Clone Corp, Huston, TX, USA), and caspase 8 (Cat. No. MBS260539, MyBioSource, San Diego, CA, USA). The findings are presented as ng/mg protein for caspases and pg/mg protein for TNF-α. Protein content was quantified using the Bradford method (Bradford 1976) using Bradford Protein assay kit (Cat.No.E-BC-K168-M, Elbascience, China).

#### Western blotting analysis

Striatal expression of p-RIPK1, p-RIPK3 and p-MLKL were assessed by the Western blot technique. Following the total striatal proteins were purely separated, 30 µg of proteins were electrophoresed on sodium dodecyl sulfate–polyacrylamide gel SDS-PAGE (10%acrylamide gel). Following the electrophoresis, using TE62 Standard Transfer Tank with Cooling Chamber (Hoefer Inc.) to relocate the protein to Hybond™ nylon membrane (GE Healthcare) then, soaked for 1 h at 25 °C in a blocking Solution (5% nonfat dry milk in blotting buffer adjusted pH to 7.4) to block nonspecific binding sites. Afterwards, the blots were soaked overnight at 4 °C in antibody solution (dilution 1:1000) against anti-p-RIPK1 antibody (Tyr284) (Cat. No. orb1629779), anti-p-RIPK3 antibody (Ser227) (Cat. No. orb1095239), anti-p-MLKL antibody (Ser125) (Cat. No. PA5-105677), and β-actin (Cat. No. ab8227). The membrane was washed many times at 25 °C for 30–60 min with blotting buffer (25 mM Tris, pH 7.4, 0.15 M NaCl, and 0.1% Tween 20), then blocked again by blocking Solution (5% nonfat dry milk in blotting buffer adjusted pH to 7.4). Next, the membrane was soaked for 1 h at 25 °C in an antibody solution containing a suitable dilution of horse radish peroxidase-conjugated secondary antibody (1.45 µg/mL). Ultimately, the intensity of the bands was assessed using a ChemiDoc™ imaging system with Image Lab™ software version 5.1. The findings are normalized to β-actin protein levels and then presented in arbitrary units.

#### Colorimetric assay

A colorimetric assay kit (ab228560) was used to determine the striatal SDH activity. Striatal cells were lysed and assayed as directed by the manufacturer’s instructions. Standard curves were used to calculate SDH activity as mU/mg protein.

### Gene enrichment analysis

Gene enrichment analysis using ShinyGO is an in-silico tool for in-depth bioinformatics analysis of gene lists, with graphical visualization of enrichment, pathway, gene characteristics and protein interactions (Ge et al. 2020). Gene enrichment analysis was achieved via ShinyGO 0.80 (http://bioinformatics.sdstate.edu/go/) to assess the association of the selected protein network including RIPK1, RIPK3, MLKL, caspase 3 (CASP3), caspase 8 (CASP8), and GFAP to relevant biological processes, cellular components, and molecular functions, as illustrated in Fig. 9A-C. All query genes are first converted to ENSEMBL gene IDs, RIPK1 (ENSRNOG00000017787), RIPK3 (ENSRNOG00000020465), MLKL (ENSRNOG00000042353), caspase-3 (ENSRNOG00000010475), caspase-8 (ENSRNOG00000012331), and GFAP (ENSRNOG00000002919). To scrutinize the interactions between the studied protein molecules, a protein-protein interactions network was constructed using STRING database and illustrated in Fig. 9D.

### Statistical analysis

We used Graph Pad Prism software (version 10; GraphPad Software, Inc., San Diego, CA, USA) to conduct all statistical analyses by utilizing the significance level of *P* value < 0.05 for all statistical tests. The findings were presented as the mean value ± standard deviation (SD). One-way analysis of variance (ANOVA) test along with Tukey’s multiple comparisons post-test was utilized to determine the significant difference between the experimental groups for all the results except for western blotting analysis and histopathological scores, Kruskal–Wallis followed by Dunn’s multiple comparison tests was used.

## Results

### Effect of NSA or MH on 3-NP-elicited body weight reduction in rats

**Fig. 2:**
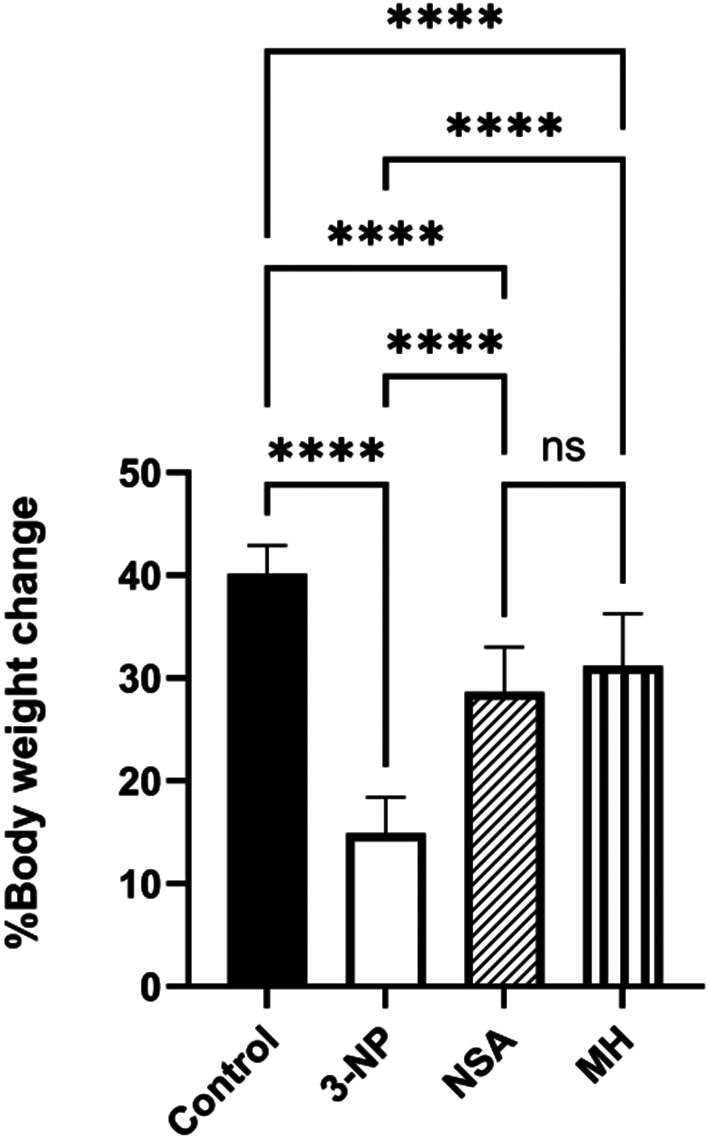
Effect of NSA or MH on 3-NP- elicited body weight reduction in rats. The results were analyzed by one-way ANOVA, then Tukey’s multiple comparisons test, and revealed as the mean ± SD (*n* = 10). Significant levels are denoted through the following: ****p < 0.0001, ns: no significance. MH, morin hydrate; 3-NP, 3-nitropropionic acid; NSA, necrosulfonamide; SD, standard deviation

Compared with the control, 3-NP injection decreased % body weight change. Conversely, protection with either MH or NSA significantly increase % body weight change (Fig. 2).

### Effect of NSA or MH on 3-NP- elicited motor and behavioral aberrations

**Fig. 3:**
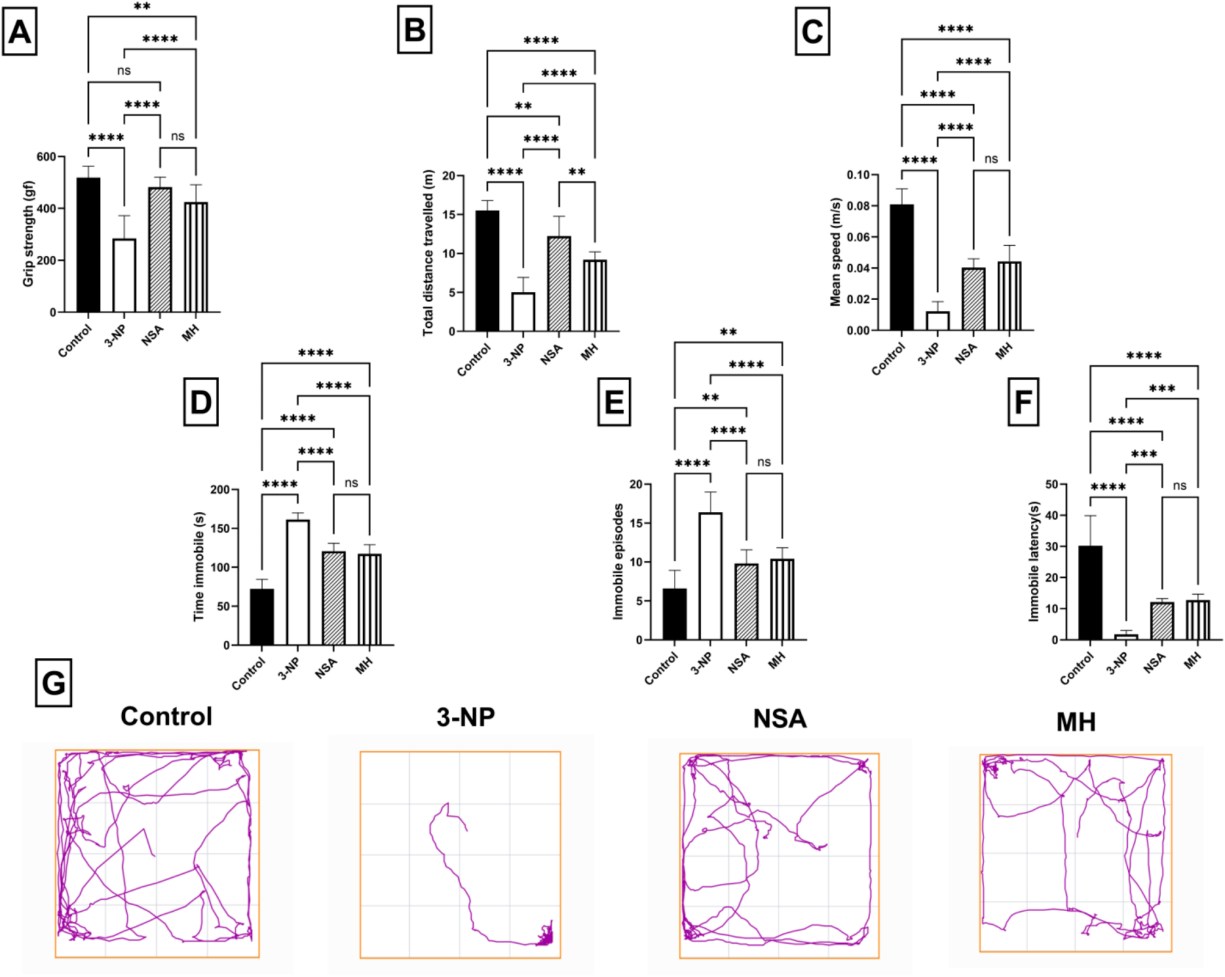
Effect of NSA or MH on 3-NP- elicited motor aberrations in the grip strength test (**A**) and behavioral aberrations in open field test (B-G) in rats. **(A)** grip strength, **(B)** total distance travelled, **(C)** mean speed, **(D)** time immobile, **(E)** immobile episodes, **(F)** immobile latency, **(G)** a representative track plot. The results were analyzed by one-way ANOVA, then Tukey’s multiple comparisons test, and revealed as the mean ± SD (*n* = 10). Significant levels are denoted through the following order: **p < 0.01, ***p < 0.001, ****p < 0.0001, ns: no significance. gf, gram force; MH, morin hydrate; 3-NP, 3-nitropropionic acid; NSA, necrosulfonamide; SD, standard deviation

3-NP rats exhibited motor and behavioral irregularities, as confirmed by grip strength, and open field tests (distance travelled, mean speed, time immobile, immobile episodes, and immobile latency). 3-NP intoxication decreased grip strength. In addition, data from the open field test showed that the 3-NP animals produced a substantial decrease in distance travelled, mean speed, and immobile latency, together with a marked increase in the time immobile and immobile episodes, in comparison to the control rats. Conversely, protection with MH or NSA prominently improved the abovementioned behavioral and motor alterations in the open field and grip strength tests (Fig. 3).

### Effect of NSA or MH on 3-NP- elicited histopathological changes

**Fig. 4:**
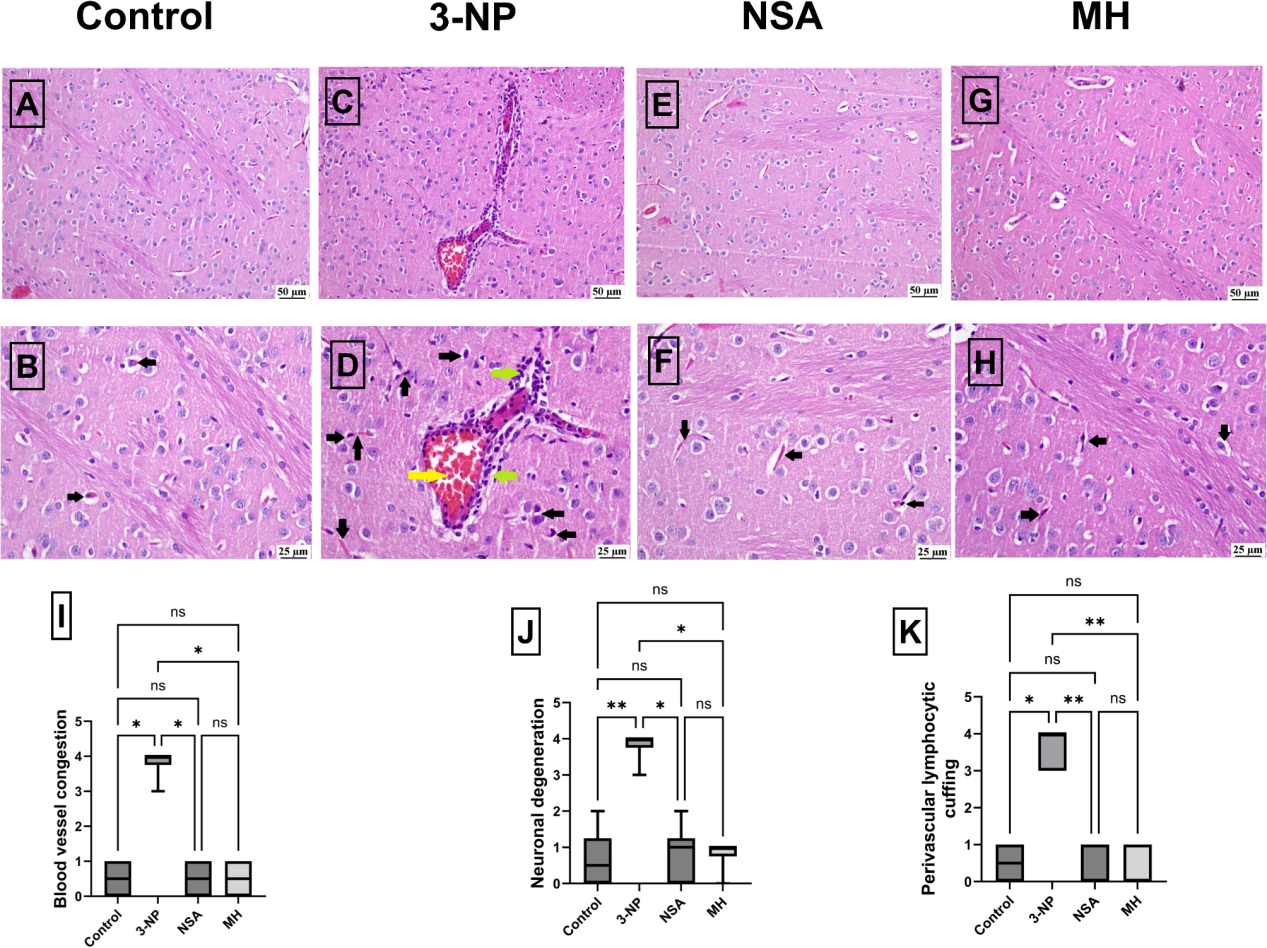
Effect of NSA or MH on histopathological alterations in the striatum region in 3-NP-treated rats. Representative photomicrographs demonstrating H&E staining of the striatum from **[A-B]** control group; normal histological appearance, **[C-D]** 3-NP group; congested blood vessel (yellow arrow) with perivascular lymphocytic cuffing (green arrow) and neuronal degeneration (black arrow)**[E-F]** NSA group; apparently normal striatum, **[G-H]** MH group; apparently normal striatum **[I]** blood vessel congestion score, **[J]** neuronal degeneration score, **[K]** perivascular lymphocytic score. The results were analyzed by one-way ANOVA, Kruskal–Wallis followed by Dunn’s multiple comparison tests, and revealed as the mean ± SD (*n* = 3). Significant levels are denoted through the following order: *p < 0.05, **p < 0.01, ns: no significance. H&E, hematoxylin and eosin; MH, morin hydrate; 3-NP, 3-nitropropionic acid; NSA, necrosulfonamide

Microscopic examination of brain sections from the control group exhibited normal striatal histology (Fig. 4A and B). The 3-NP group displayed histopathological aberrations, including severe blood vessel congestion, perivascular lymphocytic cuffing, and neuronal degeneration (Fig. 4C and D). However, in the NSA group, the striatum appeared normal with intact neurons (Fig. 4E and F). Moreover, the MH group showed a normal striatum, with nearly all examined sections showing normal striatum (Fig. 4G and H).

### Effect of NSA or MH on 3-NP-elicited changes in GFAP immunoreactivity

**Fig. 5:**
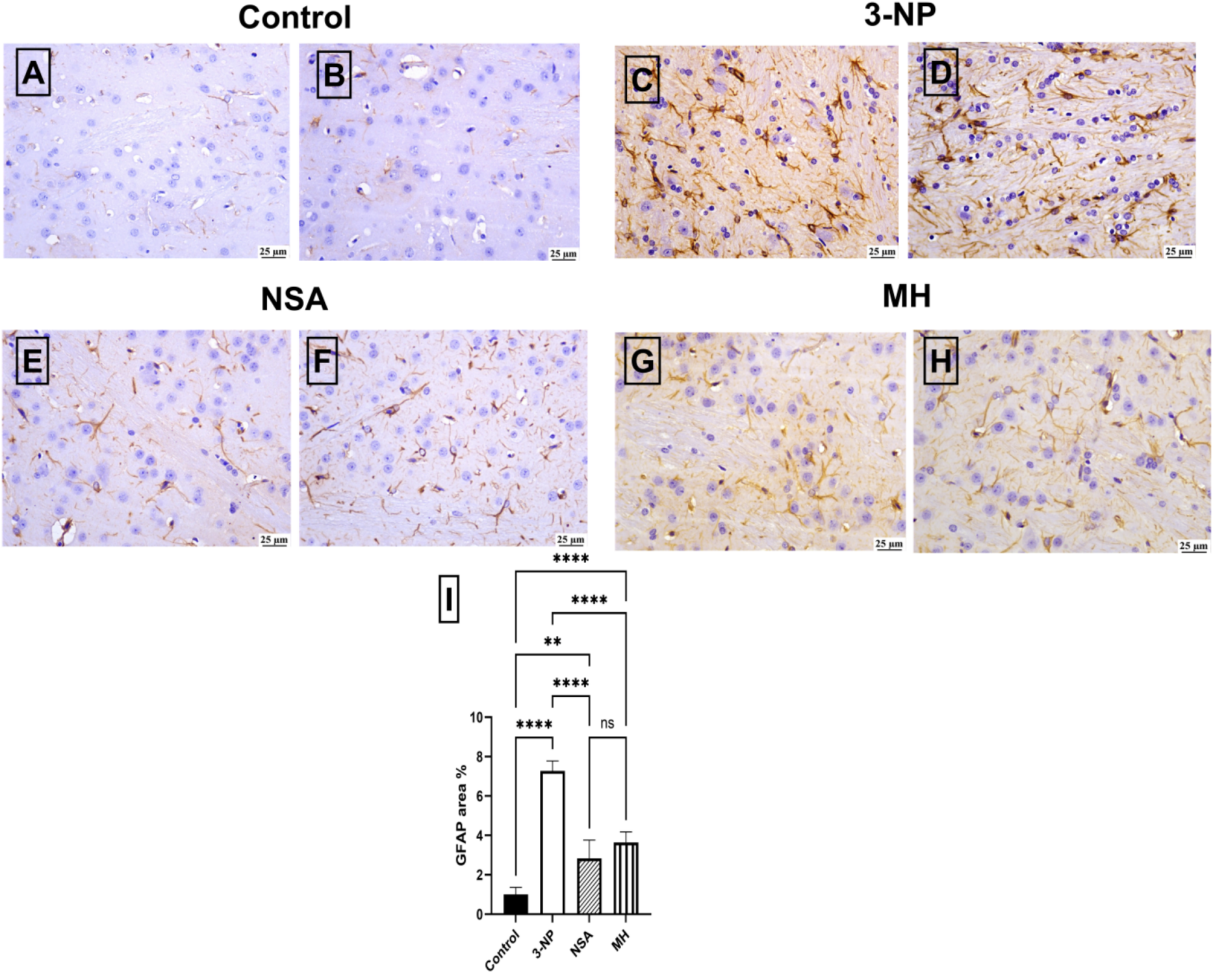
Effect of NSA or MH on striatal GFAP immunoreactivity changes in 3-NP-treated rats. Immunostaining results for GFAP in the striatum region. Photomicrographs showing weak immunohistochemical staining of GFAP in the striatum from **[A-B]** control group; weak GFAP expression, **[C-D]** 3-NP group; intense GFAP expression, **[E-F]** NSA group; moderate GFAP expression, **[G-H]** MH group; moderate GFAP expression, **[I]** Chart illustrating the quantification of GFAP area %. The results were analyzed by one-way ANOVA, then Tukey’s multiple comparisons test, and revealed as the mean ± SD (*n* = 3). Significant levels are denoted through the following order: **p < 0.01, ****p < 0.0001, ns: no significance. GFAP, glial fibrillary acidic protein; MH, morin hydrate; 3-NP, 3-nitropropionic acid; NSA, necrosulfonamide; SD, standard deviation.

The control rats exhibited weak GFAP immunoexpression in the striatum (Fig. 5A and B). Nevertheless, there was a marked elevation in GFAP expression in the 3-NP insult group (Fig. 5C and D). Moderate expression levels were detected in the NSA (Fig. 5E and F) and MH (Fig. 5G and H) groups. Compared with those in the 3-NP group, GFAP expression in the NSA and MH groups significantly decreased.

### Effect of NSA or MH on 3-NP- elicited striatal inflammation and apoptosis in rats

**Fig. 6:**
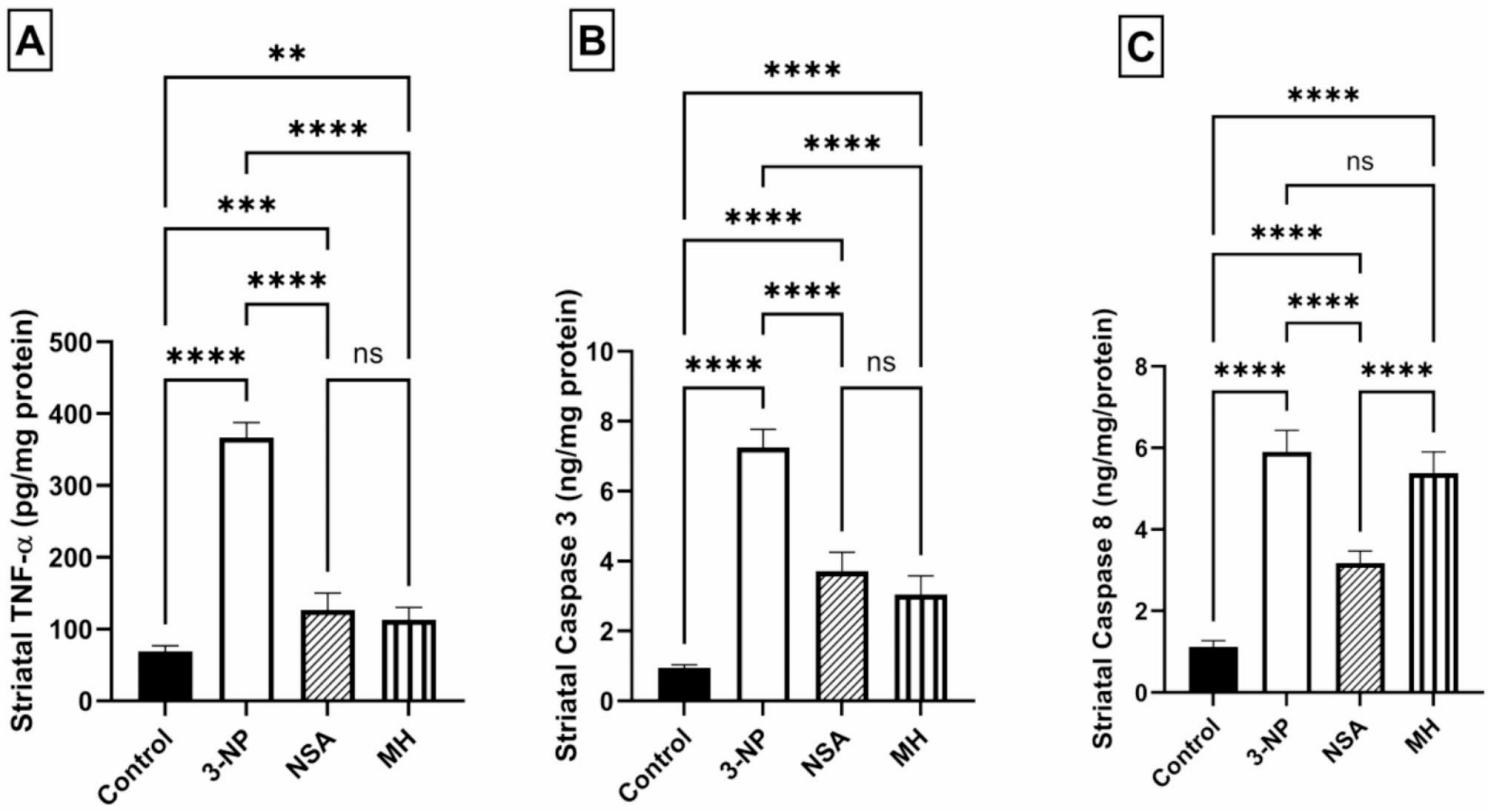
Effect of NSA or MH on 3-NP- elicited abnormalities in striatal [A] TNF-α levels, [B] caspase 3 and [C] caspase 8 activities. The results were analyzed by one-way ANOVA, then Tukey’s multiple comparisons test, and revealed as the mean ± SD (*n* = 6). Significant levels are denoted through the following order: **p < 0.01, ***p < 0.001, ****p < 0.0001, ns: no significance.MH, morin hydrate; 3-NP, 3-nitropropionic acid; NSA, necrosulfonamide; SD, standard deviation; TNF-α, tumor necrosis factor-alpha.

3-NP triggered inflammation, as evidenced by a striking 5.3-fold increase in striatal TNF-α levels in comparison to the control animals (*P* < 0.0001). On the other hand, NSA or MH administration significantly reduced TNF-α levels by 65% and 69%, respectively, relative to the 3-NP group (*P* < 0.0001). Moreover, 3-NP markedly elevated the striatal levels of caspase 3 and caspase 8 by 7.6- and 5.3-fold, respectively, compared to the control animals. On the contrary, protection with NSA significantly mitigated these elevations in striatal caspases levels, but MH affected only caspase 3 and had no effect on caspase 8 (Fig. 6).

### Effect of NSA or MH on 3-NP- elicited alteration in necroptotic markers expression in rats

**Fig. 7:**
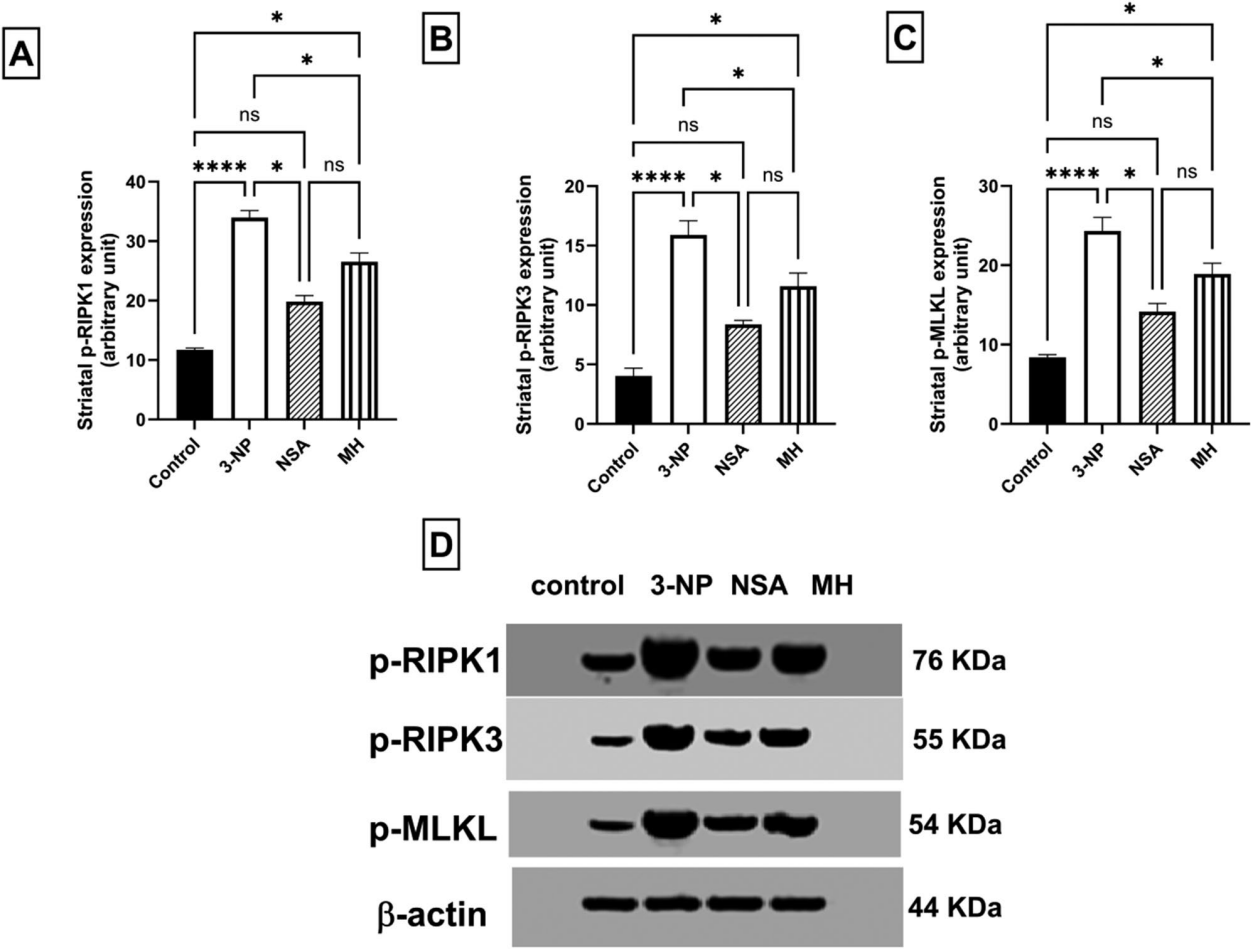
Effect of NSA or MH on 3-NP- elicited abnormalities in striatal necroptotic markers expression in rats. Expression of striatal proteins, including phosphorylated RIPK1 (Tyr284) **[A]**, phosphorylated RIPK3 (Ser227) **[B]**, and phosphorylated MLKL (Ser125) **[C]** levels. Western blot images **[D]**. The results were analyzed by one-way ANOVA, then Kruskal–Wallis followed by Dunn’s multiple comparison tests, and revealed as the mean ± SD (*n* = 3). Significant levels are denoted through the following order: *p < 0.05, ****p < 0.0001, ns: no significance. MH, morin hydrate; 3-NP, 3-nitropropionic acid; NSA, necrosulfonamide; p-RIP1/3, phosphorylated form of receptor interacting protein 1/3; p-MLKL, phosphorylated form of mixed lineage kinase domain-like protein; SD, standard deviation.

The current study demonstrated a prominent increase in the striatal levels of p-RIPK1, p-RIPK3, and p-MLKL in the 3-NP group by approximately 2.9-, 3.9-, and 2.9-fold, respectively, Relative to those in the control group (*P* < 0.0001). Nevertheless, there was a pronounced reduction in striatal p-RIPK1, p-RIPK3, and p-MLKL in NSA-treated rats by 41.5%, 47.3%, and 41.7%, respectively, and in MH-treated rats by 22%, 27%, and 22.2%, respectively, compared with those in the 3-NP rats (Fig. 7).

### Effect of NSA or MH on 3-NP- elicited aberrations in striatal SDH activity in rats

**Fig. 8:**
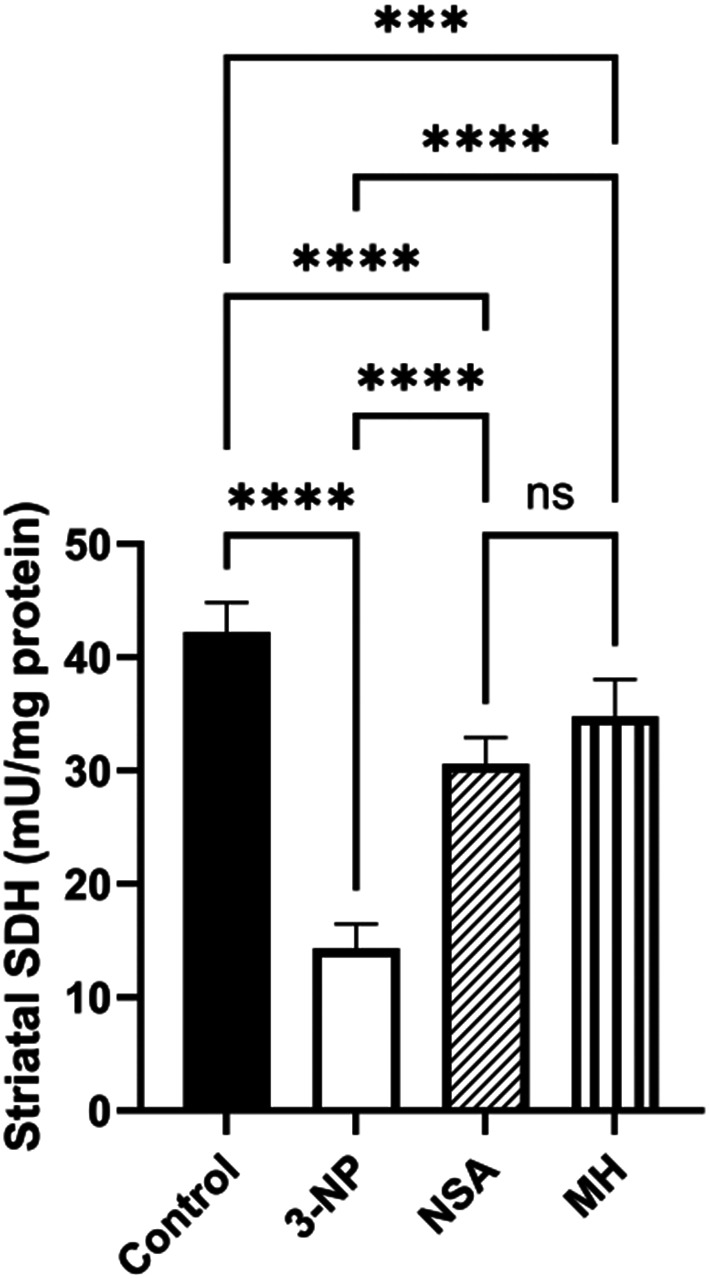
Effect of NSA or MH on 3-NP- elicited aberrations in striatal SDH activity in rats. The results were analyzed by one-way ANOVA, then Tukey’s multiple comparisons test, and revealed as the mean ± SD (*n* = 6). Significant levels are denoted through the following order: ***p < 0.001, ****p < 0.0001, ns: no significance. MH, morin hydrate; 3-NP, 3-nitropropionic acid; NSA, necrosulfonamide; SD, standard deviation; SDH, succinate dehydrogenase.

3-NP intoxication resulted in a prominent decline in the striatal SDH activity by 66% relative to the control animals. However, administration of NSA or MH caused a considerable increase in the SDH activity, by 2- and 2.4-fold, respectively, compared to those in the 3-NP group (Fig. 8).

### Gene enrichment analysis

**Fig. 9:**
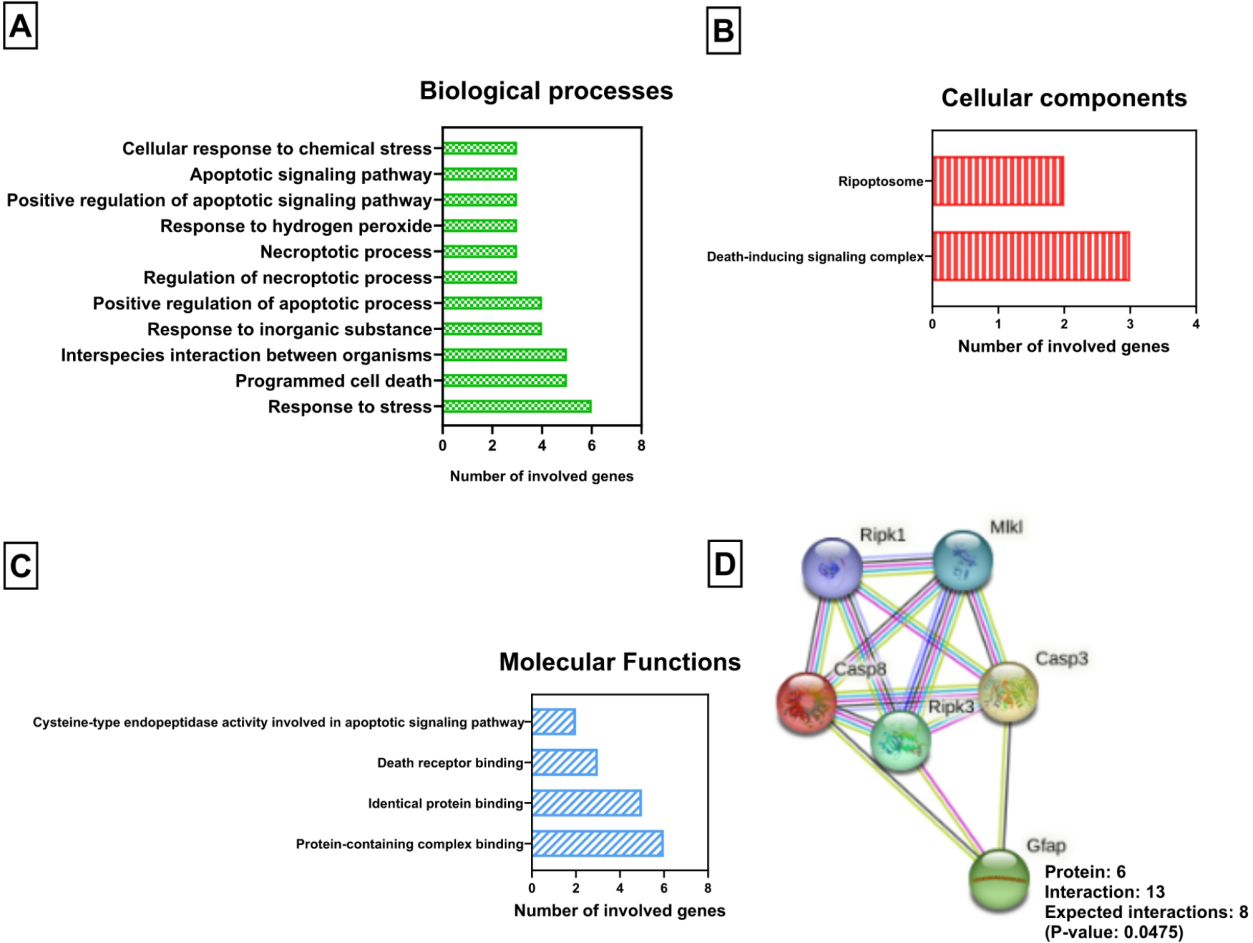
**(A-C)** STRING-enriched sets of the top pathways involving our studied genes; (**A**) biological processes domain, (**B**) cellular components, and (**C**) molecular functions. (D) A diagram illustrating the protein-protein interaction network of differentially expressed genes, generated using the STRING database. The circles denote proteins, while the straight lines denote the interactions between different proteins. Abbreviation: MLKL, mixed lineage kinase domain-like protein; CASP3, caspase 3; CASP8, caspase 8; GFAP, glial fibrillary acidic protein; and RIP1/3, receptor-interacting protein 1/3

**Fig. 10:**
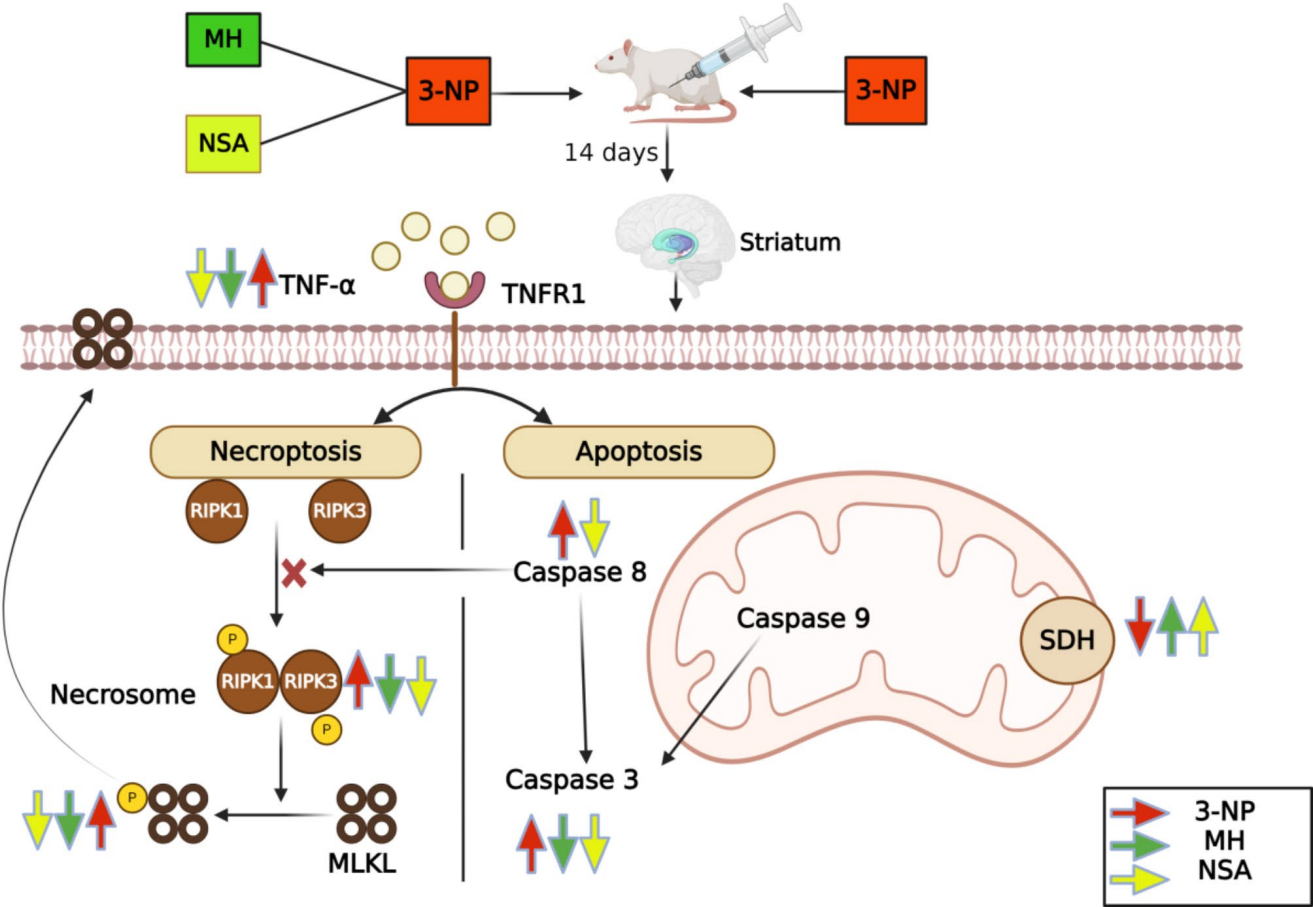
Diagrammatic illustration summarizing the neuroprotective effects of MH and NSA in 3NP-elicited HD in rat striata. MH; morin hydrate, MLKL; mixed lineage kinase domain-like protein, 3-NP; 3-nitropropionic acid, NSA; necrosulfonamide, RIPK1/3; receptor interacting protein kinase 1/3, SDH; succinate dehydrogenase, TNF-α; tumor necrosis factor-alpha, TNFR1; tumor necrosis factor receptor-1. Created with https://www.BioRender.com

Gene ontology analysis was achieved to evaluate the implication of the studied parameters to relevant biological processes, cellular components, and molecular functions. Our studied network was found to be enriched in several molecular functions. Protein-protein interaction between genes with different expressions was significant (P-value: 0.0475), deduced using STRING database (Figs. 9 and 10).

## Discussion

In our research, we investigated the neuroprotective role of MH against 3-NP-induced HD in rats. The current study confirmed the contribution of the necroptosis signaling pathway in 3-NP-mediated neurotoxicity and suggested that regulation of these signaling pathways contributes to the neuroprotective role of MH. In addition, we supported these findings by comparing MH with NSA, a known necroptosis inhibitor. The results showed that MH improved motor activity and preserved body weight. These effects were accompanied by the downregulation of p-RIPK1, p-RIPK3, and p-MLKL expressions; inhibited caspase-3 activity and TNF-α level; improved histology; and down-regulated GFAP immunoexpression in rat striatum.

3-NP, as a mitochondrial toxin, presents a robust model of HD that resembles the biochemical, pathological, and behavioral manifestations in HD patients (Túnez et al. 2010). Therefore, several studies have effectively used this model to provoke HD-like symptoms in experimental animals (Lagoa et al. 2009; Cleren et al. 2010; Elbaz et al. 2019). MH is a flavonol isolated from the *Moraceae* family and has a wide range of pharmacological properties via modulation of different signaling pathways, including apoptosis (Chen et al. 2017) and necroptosis (Abd El-Aal et al. 2022). In the current investigation, 3-NP rats exhibited marked locomotor activity deterioration, and weakening of grip strength manifested via their performance in the grip strength and open field tests. Additionally, histopathological aberrations were observed in the striatum. Moreover, 3-NP dramatically reduced body weight, which could be linked to anorexia and decreased food intake (Keene et al. 2001). These outcomes are consistent with previous findings (Ahmed et al. 2016; El-Abhar et al. 2018; Elbaz et al. 2019). Contrasted with 3-NP administration alone, pre-injection with MH diminished 3-NP-induced body weight reduction. Furthermore, MH revealed a neuroprotective action, as evidenced by improvements in locomotor activity and histopathological outcomes. Similarly, a former study reported the neuroprotective action of MH on the 3-NP-induced HD model via modulation of glutamate/calpain axis, Kidins220, and brain-derived neurotrophic factor, tropomyosin-related kinase receptor B, protein kinase B, and cAMP response element-binding protein trajectory (Mohamed et al. 2023). So far as we know, our study is the first to investigate that MH can protect the striatum from 3-NP-elicited neurotoxicity via modulation of necroptosis pathways involving RIPK1, RIPK3, and MLKL signaling. In addition, we corroborated these findings by comparing MH with the necroptosis inhibitor NSA.

TNFR1 is the primary trigger of necroptosis, which is activated via TNF-α binding (Jiao et al. 2019). This ligation induces signaling through phosphorylation and activation of RIPK1. P-RIPK1 binds to RIPK3 through their own RIPK homotypic interacting motif and formation of hetero-dimers complex called necrosome. The hetero-dimerization of RIPK1 and RIPK3 induces RIPK3 phosphorylation, which afterward induces recruit and phosphorylate MLKL, triggering its oligomerization. The oligomerized p-MLKL is translocated to the plasma membrane and creates a pore, resulting in membrane leakage, cell lysis then necroptotic cell death (Wu et al. 2020). Therefore, RIPK1, RIPK3, and MLKL are regarded as markers of necroptosis, with their overexpression and phosphorylation indicating the activation of necroptotic pathway (Wu et al. 2020). However, suppression of p-RIPK1, p-RIPK3, or p-MLKL could alleviate the neurodegenerative insults in HD. Up to now, necrostatin-1 is the only necroptosis inhibitor used in HD, and it has been shown to help sustain motor activity and body weights in the R6/2 mice while markedly delaying disease onset(Zhang et al. 2017). It was investigated that necrostatin-1 may induce apoptosis (Han et al. 2009; Jie et al. 2016). Hence, Necrostatin-1 may have harmful effects in the later stages due to distinct mechanisms involved in the early (necroptotic) and late (apoptotic) stages of HD in mice (Zhu et al. 2011). Thus, it is crucial to investigate new drugs, especially natural herbs, that can protect against HD by targeting the RIPK1/RIPK3/ MLKL pathway.

In the current investigation, we used NSA as a necroptosis inhibitor, specifically targeting MLKL. Former studies suggested that NSA can mitigate necroptosis in AD (Motawi et al. 2020), pulmonary ischemia-reperfusion injury (Ueda et al. 2022), and intracerebral hemorrhage (Zhang et al. 2022). However, as far as we know, no study has investigated the neuroprotective impact of NSA against HD. NSA reportedly affects p-MLKL levels but does not affect p-RIPK1, and p-RIPK3 levels (Jiao et al. 2019; Motawi et al. 2020). Nevertheless, other studies reported that NSA could inhibit not only p-MLKL but also p-RIPK1 and p-RIPK3 (Zhang et al. 2020). Our experimental findings demonstrate the significant effect of NSA on all necroptotic marker proteins (P-RIPK1, P-RIPK3, and P-MLKL). These findings showed that HD rats revealed robust necroptotic signaling in the striatum, declared by a significant rise in p-RIPK1, p-RIPK3, and p-MLKL expression, the protein markers of necroptosis, relative to the control animals. These results are consistent with former researches showing a correlation between the necroptosis mechanism and the progression of neurodegenerative diseases (Zhang et al. 2017). In the same context, our study investigated that MH revoked the 3-NP-induced increment in the expression of the necroptotic markers, resulting in a significant reduction in p-RIPK1, p-RIPK3, and p-MLKL levels as with NSA. Regarding this, a prior study explored MH as a necroptotic inhibitor by inhibiting the TNF-α/TNFR-1/p-RIPK-1/p-RIPK-3/p-MLKL (Abd El-Aal et al. 2022).

TNF-α is a key pro-inflammatory cytokine that promotes neuroinflammation and HD pathogenesis (Pido-Lopez et al. 2019). The elevation of the striatal level of TNF-α might be one of the probable causes explaining the improved necroptotic signaling identified in the HD. This probability is reinforced by the demonstrated essential role of TNF-α as the main inducer of necroptosis in the striatum through activation of the TNF family death domain receptor (Oliveira et al. 2018). In addition, TNF-α induces necroptosis and apoptosis signaling in HD (Pattison et al. 2006). 3-NP-induced HD is accompanied by neuroinflammation and elevated levels of different pro-inflammatory cytokines, such as TNF-α (Björkqvist et al. 2008; Hsiao et al. 2014; Moghaddam et al. 2021). Simultaneously, our results presented a prominent elevation in TNF-α in 3-NP-induced HD compared to a control group. Conversely, pretreatment with MH impeded the 3-NP-induced upsurge of the levels of TNF-α, considering its anti-inflammatory effect. This finding correlated with previous studies, showed the effect of MH on TNF-α levels in many diseases (Ma et al. 2016; Chen et al. 2017; Bachewal et al. 2018). Moreover, our results showed that NSA significantly decreased the TNF-α level relative to the HD group, which correlates with previous studies (Motawi et al. 2020; Ueda et al. 2022). Multiple hypotheses have been put forward to elucidate the pathogenesis of HD, suggesting that apoptosis initiates cell death in HD (Hickey and Chesselet 2003). Earlier study have demonstrated the triggering of caspases 1, 3, 8, and 9 and the release of cytochrome c in the striatal brain tissue of Huntington’s patients (Friedlander 2003). 3-NP induces significant apoptosis in the striatum by activating several caspases, including caspase 3 and 8 (Hickey and Chesselet 2003; Almeida et al. 2006; Moghaddam et al. 2021). It has been reported that caspase-8 can cleave the RIPK1-RIPK3 complex in the necrosome (Wu et al. 2020). Kim & Li demonstrated that necroptosis can be initiated in microglia when caspase-8 is curbed by the pan-caspase inhibitor zVAD-fmk (Kim and Li 2013). Thus, caspase 8 is connected to both necroptosis and apoptosis, acting as an extrinsic apoptosis initiator and a necroptosis suppressor (Park et al. 2021). Apoptosis and necroptosis can be activated simultaneously, as these processes are not mutually exclusive and can take place within the same organ (Linkermann and Green 2014). In the present investigation, necroptosis and apoptosis were prompted in the 3-NP-induced HD model. We observed a significant elevation in caspase 3 and 8 in HD rats compared to the control group. MH demonstrated an anti-apoptotic effect mainly through modulation of the mitochondrial-mediated apoptosis (intrinsic pathway) (Rajput et al. 2021). In the intrinsic or mitochondrial apoptotic pathway, an impaired Bax/Bcl-2 balance leads to an elevation in the level of cytochrome c, which activates caspase 3, responsible for the proteolytic degradation of proteins, thereby inducing apoptosis (Çelik et al. 2020). In earlier studies, MH reduced apoptosis by downregulating the protein expression of caspase 3 and Bax while significantly increasing the expression of Bcl-2 (Zhang et al. 2010; Chen et al. 2017; Kuzu et al. 2018; Çelik et al. 2020). These studies corroborated our findings, which showed a significant decrease in caspase 3 levels in the MH group compared to the HD group, while MH did not affect caspase 8 levels compared to the HD group. These results support that MH mainly affects the intrinsic apoptotic pathway (Rajput et al. 2021). Several investigations have reported that NSA has a protective role in necroptosis and the apoptosis pathway (Jiao et al. 2019; Menacher et al. 2019; Zhang et al. 2020). It was demonstrated that NSA could reduce the expression of caspase 3 and 8 in addition to necroptotic marker proteins (Zhang et al. 2020). In parallel, our results showed a significant reduction in caspase 3 and 8 activity in the NSA group relative to the HD group.

It has been demonstrated that HD has various mechanisms implicated in the early and late stages due to the varying sensitivity of mutant striatal cells to excitotoxicity (Graham et al. 2009). Preceding study showed that the necroptotic activation in the early disease phase could elucidate the extensive and early contribution of activated astrocytes in HD pathogenesis (Zuccato et al. 2010). Meanwhile, apoptotic characteristics were identified in the late stage (> 11 weeks) of R6/2 mice (Wu et al. 2015). Therefore, the drug that inhibits both necroptosis and apoptosis may exert a better therapeutic effect on HD (Zhang et al. 2017). In our study, MH exerted anti-necroptotic effects through modulation of p-RIPK-1/p-RIPK-3/p-MLKL and anti-apoptotic and anti-inflammatory effects via downregulation of caspase 3 and TNF-α. In addition, NSA also reduced necroptosis and apoptosis.

Post-mortem brain tissue from HD patients exhibiting neuronal loss also showed impairment in mitochondrial respiratory complexes, including complex II (SDH) (Rosenstock et al. 2012).3-NP is a suicide inhibitor for SDH enzyme in mitochondrial complex II (Túnez et al. 2010). MH is a natural bioflavonoid with antioxidant properties that help recover mitochondrial dysfunction (Rajput et al. 2021). In a prior study, morin demonstrated a protective effect on cardiac mitochondrial function by markedly increasing SDH activity and enhancing antioxidant enzyme levels(Al Numair et al. 2012). The current study aligns with these findings, showing that pretreatment with MH significantly boosted SDH activity compared with the HD group.

An upregulation of GFAP expression has been reported in HD due to heightened proliferation of astrocytes (Sorolla et al. 2012). Furthermore, 3-NP triggers gliosis as a feature of striatal degeneration, evidenced by increased GFAP immunoreactivity (Lagoa et al. 2009). The present study’s findings align with the histopathological abnormalities, as revealed by the elevated GFAP expression and degeneration of striatal neurons in the HD group. In contrast, MH and NSA significantly reduced GFAP expression and neuronal degeneration. Previous studies have demonstrated that MH has a healing role on the central nervous system by decreasing the GFAP expression levels (Kuzu et al. 2018; Çelik et al. 2020).

In conclusion, our investigation demonstrates that MH has a neuroprotective effect in 3-NP-elicited HD in rats by modulating necroptosis via targeting of the RIPK1, RIPK3, and MLKL pathway. Moreover, MH was shown to reduce neuroinflammation and apoptosis. These findings suggest that MH exerts its neuroprotective action against HD neurotoxicity through multiple mechanisms. Further clinical research is warranted to confirm MH neuroprotective effects.

## Data Availability

No datasets were generated or analysed during the current study.
